# Community dynamics influencing commercial tobacco control policy development on California American Indian lands: a qualitative analysis of baseline CAITIE data

**DOI:** 10.3389/fpubh.2025.1464022

**Published:** 2025-07-09

**Authors:** Jeremiah Wiebe-Anderson, Danielle R. Lippert, Wael K. Al-Delaimy

**Affiliations:** ^1^UC San Diego School of Public Health, San Diego, CA, United States; ^2^UC Davis Tobacco Control Evaluation Center, Davis, CA, United States

**Keywords:** American Indian and Alaska native, policy development and implementation, tobacco control, qualitative research, health disparities

## Abstract

**Introduction:**

Commercial tobacco control policies are credited with substantially decreasing the rates of commercial tobacco use among the general US population over the last few decades, yet determining the attitudes, values and beliefs that make such policies more or less appropriate for American Indian communities remains of great interest in efforts to address tobacco-related health disparities.

**Methods:**

Through the qualitative analysis of 57 baseline interviews conducted by the CAITIE project in 2021 and 2022, we explain and explore community dynamics that favor or oppose new commercial tobacco control policies in California American Indian communities.

**Results:**

The community dynamics that favor new policies include themes of changing social norms and the influence of Tribal leadership. The community dynamics that oppose new policies include respect for autonomy, preference for ‘small-p’ policies, and concern for lost revenue.

**Discussion:**

An understanding of these community dynamics may help to inform more fruitful efforts to address commercial tobacco use both within Tribal communities and in partnerships between Natives and non-Natives.

## Introduction

From time immemorial, many American Indian (AI) tribes have used traditional tobacco in a variety of cultural practices, such as personal prayers and offerings during community ceremonies. The AI tribes that engage in traditional practices consider tobacco to be not only a source of spiritual healing, but a sacred gift from the creator (1). After colonization, these traditional tobacco practices were prohibited by the US government until the passage of the American Indian Religious Freedom Act in 1978. During this prohibition and into the present, Big Tobacco aggressively marketed to and campaigned for favorable public relations in Tribal communities through such exploitative strategies as using American Indian imagery and iconography and specific sales promotions in AI communities (1–3). As a result, commercial tobacco became the sole point of practical access to tobacco for many AI tribes. Although traditional tobacco was and continues to be cultivated and used by some CA tribes, a problematic shift occurred in many tribes where commercial tobacco supplanted traditional tobacco. What had been a sacred medicine became a source of sickness. What had been a gift from the creator became leverage in the pursuit of corporate profits.

Commercial tobacco products are used by American Indians (AIs) at disproportionately high rates, compared to other racial/ethnic demographics, with estimates of prevalence of use among AIs ranging from 31.8 to 38.9% (4–7). The national incidence of mortality among AIs attributable to tobacco use is double that of any other demographic group in the United States (4, 7). Although significant progress has been made over the last several decades in reducing commercial tobacco use among the general population, during this same period, the aggregate AI prevalence of commercial tobacco use has stayed relatively steady (6, 8, 9). Tobacco Control policies, such as smoke-free air regulations and excise taxes on commercial tobacco products, are largely responsible for the decline in smoking rates seen in the general population (9–11). In their Framework Convention on Tobacco Control, the World Health Organization defines tobacco control as “a range of supply, demand and harm reduction strategies that aim to improve the health of a population by eliminating or reducing their consumption of tobacco products and exposure to tobacco smoke” (12). We specify *commercial* tobacco control (CTC) policies explicitly in this work to pay respect to the traditional tobacco practices of AIs and to highlight that the tobacco control policies discussed in this context are not in reference to those traditional tobacco practices.

While the prevalence of smoking commercial tobacco among AIs may exceed the national smoking prevalence in aggregate, this obscures the reality of Tribal diversity. The prevalence of commercial tobacco use in AIs vary dramatically by region (4, 5, 13). Likewise, the disparities in health outcomes also vary across regions (13, 14). For California AIs particularly, the prevalence of commercial tobacco use has declined substantially in recent years, dropping from 36.5% in 2003 to 18.5% in 2021, yet still remain relatively high compared to the prevalence of smoking in California’s overall adult population (15, 16). Clearly, historical and cultural differences between tribes and across regions have left measurable effects on the prevalence of smoking.

AIs are often aggregated in research and treated as a homogeneous group, which too commonly leads to inappropriate, ineffective public health interventions. Commercial tobacco control (CTC) interventions designed by and for non-indigenous peoples are less effective at addressing commercial tobacco-related health inequities in AI communities (1, 17). Therefore, public health practitioners must be cautious not to apply implicit assumptions and stereotypical thinking when crafting possible interventions. What works for CTC in one tribe may not be appropriate for another and although there may be some overlap in AI tribes’ preferred methods of CTC, a one-size-fits-all approach to CTC is not the desired goal (1, 18).

AI tribes across the nation have expressed similar priorities regarding CTC efforts, such as locally-tailored language, distinguishing traditional AI tobacco practices from commercial uses, responsibility to family, centering elder wisdom and protecting future generations (1, 19–21). Despite the similarities in expressed priorities for CTC, the AIs highlighted in these articles have implemented, or have chosen not to implement a variety of their own adaptations of CTC policies. This paper intends to describe and explore the community dynamics that influence the development of commercial tobacco control policies on California AI lands among a diverse group of tribes.

## Methods and materials

The California American Indian Tobacco Initiative Evaluation (CAITIE) team has been tasked with the independent evaluation of the programs resulting from the California Department of Public Health, California Tobacco Prevention Program (CTPP) American Indian Initiative to Reduce Tobacco-Related Disparities. The American Indian Initiative entails state grants funding used by California American Indian tribes and the community organizations that support them for the development of self-directed efforts to reduce commercial tobacco use within their respective communities, specifically policies focusing on reducing exposure to secondhand smoke, tobacco smoke residue, tobacco waste, and other tobacco products. The role of the CAITIE team was to interview, survey and otherwise collect data from designated key informants (KIs) with first-hand knowledge of the grantees’ activities, in order to assess what is working well with these efforts, where successes are being realized and where obstacles occur. This data collection was to be conducted at two to three points in time.

### Sampling

The process of selecting and interviewing KIs is part of the evaluation approach agreed upon by the CTPP, the funded Tribal leadership and the community organizations serving the participating tribes, and the University of California San Diego (UCSD). This sampling process relied upon purposive sampling, which involved a memorandum of understanding whereby the process of engaging the communities is outlined as follows: The Director and staff of the funded AI project nominated KIs who are known by the community and are from the community after seeking their approval for participation. Their contacts are then given to the UCSD team, who reach out to them for approval, consent, and an interview date. Every funded project associated with an individual Tribe agreed to nominate three KIs for interviews, while the community organizations serving multiple tribes were requested to nominate six KIs for interviews. As such, the sampling protocol for this research relied upon purposive selection of KIs on behalf of the participating Tribes and community organizations. Program staff were connected to the nominated KIs and were not part of this selection process and thus depended upon the participating Tribes and community organizations to nominate KIs who were knowledgeable about the topic at hand. All KIs were provided with a description of the research design and aims prior to participation, and informed consent was acquired verbally before the interviews were conducted and before recording began.

Ultimately, 57 individual KIs were interviewed for the analysis in this manuscript. They represented all of the 12 tribes and four American Indian serving community organizations who were funded through the program. The 12 tribes included the Pala Band of Mission Indians, the Picayune Rancheria of the Chukchansi Indians, the Toulumne Band of Me-Wuk Indians, the Enterprise Rancheria of Maidu Indians of California, the Hopland Band of Pomo Indians, the La Jolla Band of Luiseño Indians, the Rincon San Luiseño Band of Mission Indians, the San Pasqual Band of Mission Indians, the Yurok Tribe of the Yurok Reservation, the Coyote Valley Band of Pomo Indians, the Redwood Valley Little River Band of Pomo Indians, and the Sherwood Valley Band of Pomo Indians. The four American Indian serving community organizations included the California Indian Museum and Cultural Center, the Lake County Tribal Health Consortium, the Native Star Foundation, and United Indian Health Services. All interviews were conducted via Zoom and transcribed using the Otter AI transcription program, then manually reviewed and edited by CAITIE team members for accuracy. The transcript data was then entered into Dedoose Qualitative Data Analysis Software for analyses and coding.

This dedicated qualitative analysis of baseline CAITIE interview data is nested in the larger work of the CAITIE Project. This paper aims to answer the following research questions:

1. What Tribal community dynamics favor new CTC policies?
2. What Tribal community dynamics oppose new CTC policies?

In the CAITIE project’s key informant interview script, there are three open-ended questions that prompt the KIs to speak about CTC policies, their enforcement and how they are perceived by the community. Question #1 asks broadly about the efforts of grant-funded programs. It asks: “What activities and policies are happening as a result of this American Indian Initiative program?” Question #9 asks: “In what ways has the community shown readiness and public support to implement commercial tobacco policy changes?” If the KI asks about ‘readiness’ or provides a short answer, the interviewer employs this follow-up question: “Can you tell me about how the community has shown it is ready to take action regarding tobacco use?” Question #13 asks: “How are tobacco Tribal codes and policies enforced?” After the KI finishes their response, the interview has a required follow-up question, which asks: “In general, what happens to those who do not follow a tobacco Tribal code or policy?” These three questions (and their respective follow up questions) represent those that were most directly related to the focus of the present study. All questions asked of all KIs were standardized and presented in the same manner to all KIs.

This policy-focused qualitative analysis of the baseline CAITIE interview data employed values coding and grounded theory to answer these research questions. Through these frameworks, the beliefs, attitudes and values of the KIs have been evaluated for emotional/attitudinal valence and coded. Each theme of beliefs, attitudes and values needed to be mentioned at least 10 times in the baseline data in order for it to reach the threshold of warranting its own code. One of the program staff (the first listed author of the present manuscript) was the sole, dedicated coder for this work, and was responsible for reviewing transcript data for accuracy against the interview recordings, and for utilizing Dedoose Qualitative Data Analysis Software for analyses and coding. The orientation of this paper is not toward exposing CTC deficits, but rather, toward building a better understanding of what influences the development of CTC policies on California AI.

## Results

Out of 57 baseline interviews conducted with KIs on behalf of our community partners (grantees), 31 KIs (54.4%) were Tribal members, but not program staff, eight (14%) were both Tribal members and program staff, and 18 (31.6%) were program staff, but not Tribal members. From this baseline interview data, five themes related to community dynamics and CTC policy development were identified and labeled. These themes are explored in two sections. The first section, ‘Tribal community dynamics favoring new CTC policies’, includes the themes of changing social norms and the influence of Tribal leadership. The remaining themes described in the second section, ‘Tribal community dynamics opposing new CTC policies’, include the themes of respect for autonomy, preference for ‘small-p’ policies (i.e., suggestive not punitive policy), and concern for lost revenue.

### Tribal community dynamics favoring new CTC policies

Tribal community dynamics favoring new CTC policies and their enforcement can come in the form of many different behaviors or demonstrations of values, beliefs and attitudes. This section describes and explores two themes: changing social norms and the influence of Tribal leadership.

#### Changing social norms

There are many aspects of the American Indian Initiative that contribute to changing social norms surrounding commercial tobacco use on CA AI lands. This is not limited to policy change, by any means. However, CTC policy change appears to offer a key point of establishing expectations, which can, over time, change social norms. Throughout the baseline data, the theme of changing social norms was expressed in 21 separate occasions. Here, one KI speaks directly to the confluence of the goals and motivations for policy change and the belief in the power of policy to change social norms:

*"… we have projects where we try to chip away at the use of tobacco products, and then also prevent more and more people every year from taking that on. And getting rid of it in our housing is, I think, probably a really key way because we may have a fight or grumblings about it at the beginning, but over time, it will shift social norms, hopefully … the more places that we're encouraging and expecting people to not use tobacco products, that will slowly get rid of it, hopefully."*

Here the KI, who is a Tribal member and not program staff, clearly expresses the belief that new CTC policies have a role to play in changing the social norms within their respective community. However, there are a number of other important points to unpack. This excerpt begins with the understanding that changing social norms, perhaps especially through new CTC policies will take time. They say “… where we try to chip away …” suggesting that changing social norms is an undertaking that requires sustained effort. Implementing new CTC policies and changing social norms is an incremental process. They say that “… we may have a fight or grumblings about it …” meaning that it is unrealistic to expect that any new policies will be met with unanimous support. Even if the majority of Tribal members approve of a new CTC policy change, disagreement is to be expected and dissenting voices should be heard.

The KI concludes this statement with what is, ultimately, a reiteration of the role that CTC policies (specifically smoke-free policies) can play in changing social norms. They say “*… the more places that we are encouraging and expecting people to not use tobacco products, that will slowly get rid of it …*” This statement is significant because it speaks to the role of policies in shaping social norms and guiding behavior change through expectation setting, as opposed to their punitive actions following policy violations, such as citations or Tribal court appointments. While punitive actions may still play an important role in curtailing commercial tobacco use on CA AI lands, they remain a source of resistance to new CTC policies. As this KI is a Tribal member themselves, they have an insider’s perspective on the existing social norms, which lends credence to their belief that policies that set expectations can play a role changing social norms.

Another example of this theme can be seen in the following excerpt from an interview with a KI who is a Tribal member, but not program staff:

*Interviewer: "What’s the most significant impact [of the program]?”*

*KI: "I would just say, the awareness that this is something that the Tribe is taking seriously and wants to address for our community."*

Here the KI states how they regard the community’s increasing awareness of how seriously their tribe is taking the health hazard of commercial tobacco as the most significant impact of the program. They add that this is something that the tribe “… wants to address …” Together these sentiments of increasing awareness of the problem and a desire to act are emblematic of the theme of changing social norms regarding commercial tobacco and its regulation on Tribal lands. Keeping in mind that this KI is a Tribal member themselves, and not program staff, the awareness they speak of is owned by the community, and the desire of the Tribal leadership to act is seen as impactful, perhaps then affecting the prevailing social norms.

#### The influence of tribal leadership

The theme of the influence of Tribal leadership was expressed 38 times in the baseline data. The following excerpt is one of many examples that provide evidence for the influence of Tribal leadership as a community dynamic that can favor new CTC policies:

*Interviewer: "In what ways has the community shown readiness and public support to implement commercial tobacco policy changes?"*

*KI: “Well, we have a young Tribal council and I don’t believe one of them smokes. So we have their backing when we want to put out a Tribal policy.. They’re going to help us get that in place a lot faster than the other older Tribal council because all of them smoke.”*

This KI, who is not a Tribal member, but is program staff, makes a statement here that captures the influence of Tribal leadership. The newer, younger Tribal council members do not smoke and are actively helpful when program staff approach them about CTC policy changes. In contrast, we can see that the KI knew the older Tribal council members to be resistant to CTC policy changes, perhaps in part because of their own affinity for smoking commercial tobacco. As this excerpt demonstrates, the influence of Tribal leadership can cut both ways - either adding momentum to policy change efforts or hindering them, and as this KI is not a Tribal member themselves, but is program staff, we can infer from this statement that the program must be sensitive to the whims of the Tribal leadership.

Here the theme of the influence of Tribal leadership also appears to be synergistic with the theme of changing social norms. Younger Tribal council members smoke commercial tobacco less frequently, whereas the presumption is that, by contrast, the previous, older Tribal council members were more prone to smoking commercial tobacco. It appears that leaders who defy policies can set a tone of dismissing policies for the community broadly. Alternatively, other leaders (typically younger) may choose to establish new social norms and exert their influence in favor of new policies. In this regard, changing social norms appears generational, rather than necessarily a result of the American Indian Initiative. Additionally, program staff working on CTC policy change have also expressed how crucial access to and relationships with Tribal leadership can be for the success of their efforts, lending further credence to this theme and its impact on CTC policy development.

Another instance of the theme of the influence of Tribal leadership can be seen in the following excerpt:

*"… people listen to them and the community has respect and listen to a lot of these organizations and leaders. And they have a lot to say and it goes a long way."*

Here the KI, who is a Tribal member, but not program staff, speaks to the sway that Tribal leadership has over the opinions of the Tribal members who “… respect and listen …” to them. In this way, Tribal leaders can set the tone for how the tribe receives new CTC policy recommendations, or if new policy recommendations are meaningfully considered at all. For a Tribal member who is not program staff to acknowledge that the influence of Tribal leadership, “… goes a long way,” suggests that they may have seem this influence at play in other Tribal matters as well, further supporting the relevance of this theme. These are just two examples of 38, which demonstrate the influence of Tribal leadership on CTC policy development.

### Tribal community dynamics opposing new CTC policies

Tribal community dynamics opposing new CTC policies and their enforcement can come in the form of many different behaviors or demonstrations of values, beliefs and attitudes. This third thematic category covers the themes of respect for autonomy, preference for ‘small-p’ policies, and concern for lost revenue.

#### Respect for autonomy

The theme of respect for autonomy was expressed by the KIs 11 times throughout our baseline interviews. This next excerpt highlights a particular community dynamic in which Tribal members both acknowledge the harms of commercial tobacco products and, at the same time, resist new smoke-free policies. The resistance appears to be predicated on the value of respect for autonomy.

*“We did one small survey and most people here completely agree that smoking is bad … but then almost everybody said that they will not want to live somewhere that had.. smoke-free policy. So that kind of blew our mind that even the nonsmoker said that they wouldn't want to live somewhere where there was a policy where you couldn't smoke. And again, I think that's because people were thinking that we're going to be taking away rights or some sort of autonomy.”*

In this excerpt, the KI, who is program staff, but not a Tribal member, identifies a disconnect between having knowledge of the harms caused by commercial tobacco and opposing smoke-free policy. Even the non-smoking survey respondents indicated this position. Despite knowing that smoking commercial tobacco causes harm, they seem to be opposed to restrictive policies in principle. Through this lens, opposing restrictive CTC policies can be seen as preserving autonomy. As this KI is not a Tribal member, but is program staff, we can see that they are trying to make sense of this apparent disconnect here, and their conclusion lands squarely on the high value this Tribal community places on autonomy.

This theme is also captured in this next quote from a KI who is both a Tribal member and program staff:

*"… we wanna protect ideas, we wanna protect the elders, but they’re also very conscientious about, you know, people’s choices … it’s really up to the community members … I think there’s a little bit of apprehension about whether people, you know, will go for that, and whether we could limit where people can smoke …"*

In this quote, the KI clearly states that the program is sensitive to the Tribal members’ individual preferences regarding CTC. In emphasizing the choices of the people and how “… it’s really up to the community members …” the KI reveals that there is strong doubt about whether Tribal members would accept limitations on their freedom to smoke where they choose. This apprehension toward smoke-free policies reflects the deeply held Native value of respect for autonomy, and can be seen in a larger context as community dynamic that may act in opposition to any restrictive CTC policies. As this KI is both a Tribal member and program staff, we can extrapolate that they both want to uphold the values of their Tribal community while also pursuing the goals of the program, which is to reduce commercial tobacco use. It is a fine line that they walk as they hold both of these truths simultaneously. The result is an acknowledgment of doubt that hard limitations on where people can smoke fail to respect the autonomy of the community.

#### Preference for ‘small-p’ policies

A small-p policy is a policy that is not enforced through punitive measures, such as citations or court appointments, whether or not those punitive measures for enforcement are written into the language of the policy. Small-p policies operate by establishing social expectations and are enforced through mutual accountability. In this way, we say that small-p polices are suggestive, rather than punitive. The theme of preference for ‘small-p’ policies was articulated 27 times by KIs.

This next excerpt illustrates how one KI, who is a Tribal member but not program staff, understands the nature of smoke-free policy enforcement in their reservation:

*Interviewer: "So like in the communal areas in general, what happens to those that don't follow that [smoke-free] policy?”*

*KI: “Oh, they're just usually asked to not smoke."*

Here the KI states plainly that the typical response to non-compliance with smoke-free policies is simply a verbal reminder. Even when enforcement mechanisms are in place, such as citations or Tribal court appointments, the predominant standard is to treat all policies as suggestive, rather than directive. As this KI is a Tribal member, but not program staff, we can read from their concise answer that they have seen this response personally. This sentiment, broadly speaking, was the same one expressed by all other KIs who spoke on this theme.

This next quote from a KI who is program staff but not a Tribal member captures the crux of this theme of preference for ‘small-p’ policies:

*"… when someone from the outside is trying to place policy on them, it can seem not intimidating, but almost disrespectful, and they don’t really communicate with that very well. So I’ve been trying to change the language within our activities to not make it a policy, but make it you know, like a pledge or voluntary …"*

As this KI is program staff, but not a Tribal member, their goal of crafting policies that are voluntary is emblematic of their recognition of the preference for small-p policies.

Another KI, who is program staff but not a Tribal member, spoke to the difficulty not just in enacting policies, but also in making them enforceable. They state:

*"I think, you know, passing the policies, probably not as hard as enforcing the policy."*

When a policy does not have enforcement, or is rarely if ever enforced, it means it functions more as a suggestion than a directive; participation in such a ‘policy’ would be entirely voluntary. However, that may not, after all, be such a bad thing. As was discussed earlier, the power of changing social norms should not be underestimated. If a policy is established such that it is meant to set expectations, without a paternalistic threat of punitive action for non-compliance, perhaps that is enough to foster changing social norms. Perhaps mutual accountability is sufficient for the purposes of reducing commercial tobacco use in a given CA AI community, as this is a common tenant in Tribal culture. Either way, a suggestive policy that passes would, presumably, be more impactful than an authoritative capital-P Policy which never passes. While the KIs did not use that explicit language of ‘small-p’ policies, many spoke of policies that had no discernable enforcement mechanisms. These included such program efforts as voluntary smoke-free housing pledges (mentioned by seven KIs) and culturally appropriate smoking cessation programs (mentioned by five KIs). Other approaches to suggestive commercial tobacco control may include decisions to allocate more funding for youth education, or for the organization of talking circles to rekindle traditional tobacco practices and spread awareness about how those practices differ from commercial tobacco use.

#### Concern for lost revenue

The theme of concern for lost revenue and other related attitudes generally arose in regards to prohibiting the sales of certain products in Tribally-owned businesses, or through making casinos smoke-free and was mentioned 15 times in the baseline data. After having just discussed the banning of vaping product sales in their tribe’s convenience store, as well as the recent passage of excise taxes on the sales of commercial tobacco products and a smoke-free policy for their casino, a KI, who is a Tribal member, but not program staff, is asked about the possibility of prohibiting all sales of commercial tobacco products in their tribe’s stores. This is their response:

*"… I don’t see that happening in the near future."*

Despite the considerable CTC policy progress indicated by this KI, they were skeptical that a total ban on commercial tobacco products would be feasible at this time. Although some revenue is lost from bans on other products, or from excise taxes, it appears that a total ban on commercial tobacco products is too far, at least for now. This speaks to the potential limitations of CTC policies where they conflict with sources of revenue, even in communities that are otherwise receptive.

Several other KIs discussed the prospect of smoke-free gaming/casinos and these discussions were usually accompanied by descriptions of community dynamics opposing new CTC policies, like respect for autonomy and concern for lost revenue. The obstacle here is not just that new policies may infringe on the autonomy of casino goers, but there is a perception that there may be fewer casino goers if smoking is prohibited, risking reduced revenue to the tribe. These concerns appeared to be closely associated with Tribal council members and administrators in gaming departments, as can be seen in this quote from a KI who is a Tribal member but not program staff:

*"And, you know, trying to encourage the decision makers who determine the economic profit of any kind of business, that [Tribal gaming going smoke-free] may not be worth it."*

Being a Tribal member, but not program staff, this KI can be understood to be speaking to this issue from an insider’s perspective, such that their ideas of what is and is not ‘worth it’ to Tribal decision makers is not mediated by externally-funded programming goals. However, according to the informal feedback from several collaborators and community members, the COVID pandemic introduced a novel motivation for smoke-free policy enactment in Tribal casinos, as Tribal leadership understood that smokers were at a higher risk of complications of COVID. Some Tribal governments leveraged that knowledge to implement and enforce new smoke-free policies in their gaming departments, although most of these were temporary.

## Discussion

The Tribal community dynamics related to CTC development on CA AI lands described and explored in this paper may be useful to California AIs who seek to implement CTC policies in their communities. These findings may also serve non-Native researchers, health professionals and state officials as they endeavor to partner with AI communities. In this regard, the Tribal community dynamics that oppose new CTC policies may be especially significant for non-Native partners to understand. Without such an understanding, the commonly-held Native value of respect for autonomy may not be adequately considered, small-p policies may not be properly centered in CTC efforts and the concern for lost revenue may not be appropriately addressed. Such failures on behalf of non-Native partners would likely result in unnecessarily contentious and less productive partnerships with AI communities.

The implications of the present study are supported by the research of Lee, Smith and Thompson, which states that “capacity building of non-Indigenous researchers, to conduct research with Indigenous peoples in ways that uphold core principles and values, is needed” (17). We concur that non-Indigenous researchers and public health professionals working with AI communities to address the health disparities related to commercial tobacco use must endeavor to uphold the core principles and values of those they are in partnership with. Furthermore, our findings related to preference for small-p policies (elsewhere referred to as voluntary policies or alternatives to mainstream interventions), concern for lost revenue, changing social norms and the influence of Tribal leadership are corroborated by previous studies on commercial tobacco research and interventions in other AI contexts (17, 20, 21).

The present study is also comparable to the California Department of Public Health-funded project by Soto and Moerner, which assessed the readiness of 12 California Tribal communities to work on commercial tobacco-related policy (22). The resulting report corroborates the conclusions of the present study in at least two ways: they determined that there was a sizeable preference for small-p policies and that there was concern for fear of lost revenue should Tribal gaming go smoke-free. This corroboration suggests that the similar findings of the present study are reliable.

However, the conclusions of Soto and Moerner differ from those of the present study in three ways. The first is that Soto and Moerner’s study found that there was no support or undetermined support for universal smoke-free policies covering workplaces, public spaces, businesses and casinos. Although the work done by CAITIE does not approach smoke–free policies in such a comprehensive manner, the public support and readiness for smoke-free policies in each of these locations separately appeared substantially higher in our analysis, even for Tribal casinos. It is possible that this difference can be accounted for by how smoke-free policies were framed in the Soto and Moerner study, as grouping all smoke-free policies together may have resulted in lower perceived smoke-free policy readiness among participants. The sampling and selection of key informants for both projects may also factor into this difference. Although both projects relied on purposive sampling, ours was a non-random sample from 12 individual tribes and four AI-serving community organizations that had actively applied for and were utilizing grant money to reduce commercial tobacco use, thus suggesting some bias toward interest in CTC. Time may also be a factor here, as Soto and Moerner published in 2021, while our data collection was just beginning at that time, and social norms surrounding CTC may have continued to change during this interval.

The second difference between the conclusions of the present study and those found in the report by Soto and Moerner, is that their study found that a majority of participants stated that the smoke-free policies in place were strictly enforced. With the exception of the smoke-free policies of Tribal government offices and buildings, our analysis indicated a much lower confidence in the strict enforcement of existing smoke-free policies, reflecting the themes of respect for autonomy and preference for small-p policies. The third contrasting feature is that Soto and Moerner’s report found that a majority of key informants would support Tobacco 21 laws specifically. While the analysis for the present study did find mentions of Tobacco 21 age limits for commercial sales, these were not particularly prevalent. This is suggestive of Tribal heterogeneity and the limited generalizability of studies of this kind. Despite these differences, the present study and the report by Soto and Moerner stand out as the only studies of their kind to date, in describing and exploring the current state of the attitudes, values and beliefs that shape CTC policy development in modern, California AI communities.

On the matter of concern for lost revenue, a majority of the studies which analyzed the economic impacts of casinos becoming smoke-free conclude that there is no statistically significant loss in patronage or casino-generated revenue following smoke-free policy implementation (23–26). The findings of the present study can help to prepare both Native and non-Native CTC policy advocates to anticipate sources of community resistance, and should be cautious not to dismiss concern for lost revenue outright. Rather, the conclusions of these economic analyses can be shared to assuage that concern respectfully.

Lastly, it is worth reiterating that the present study does not produce and is not intended to produce specific policy recommendations or actions for all AI Tribes or communities in California. While the 57 KIs representing the 12 Tribes and four community organizations that participated in this research offered many insights indicating that certain CTC policies that may be more or less agreeable to AIs in California, there are 109 federally recognized AI Tribes, and an additional 65 unrecognized Tribes (27). Furthermore, there is tremendous variability in the attitudes, values and beliefs across and within these different Tribes, and as such, any attempts to make sweeping policy recommendations is not only unproductive, but disrespectful to the reality of Tribal heterogeneity. Rather, this work serves to support and inform future CTC policy research and development, particularly cross-cultural collaboration to those ends.

### Limitations

One limitation of this study is that the primary analyst and author of this work is non-Native and may have inadvertently misrepresented the data. Two of the co-authors (Chag Lowry and Danielle Lippert) are California American Indians who have offered their feedback in order to minimize this limitation to the extent possible. Another limitation is that the data available for this qualitative analysis is strictly from baseline/first wave interviews. One or more waves of data collection are planned, or are currently underway, which will likely furnish pertinent information. It should also be reiterated that the sharp distinction of one theme from another can, at times, be somewhat blurred and the boundaries separating them are more meant to facilitate the accessibility of this work, and less to establish strict differences between the thematic categories. The influence of Tribal leadership, for instance, is a community dynamic that can cut both ways, but because of the synergistic effect this theme demonstrated with changing social norms, it was appropriate to address this dynamic with the others in favor of CTC policy change. Another limitation of this study is that due to Tribal heterogeneity, these findings should not be considered representative of all CA AI tribes.

## Conclusion

Our analysis explores the nuanced and delicate social and historical context of tobacco use among American Indians in California. Mainstream approaches to CTC might not be as effective or appropriate in AI communities for the reasons described in this manuscript. Specifically, there appears to be a reluctance to enforce policies with penalties, versus suggestive restrictions, which we refer to as small-p policies. The sacred use of tobacco historically, its prohibition, and the targeted promotion and public relation campaigns by tobacco industries in AI communities, each introduced further complexity for CTC policy development and enforcement. We found that younger generations of AIs generally have different attitudes about commercial tobacco use and were contributing to changing social norms. They voiced more support for CTC policies and for ceremonial use of tobacco (keeping it sacred), rather than commercial tobacco use. There is much to learn from the diversity of AI peoples from across the state of California that may be relevant to CTC efforts in other indigenous populations nationally and globally. However, we maintain that a one-size-fits-all approach to CTC is inappropriate in this context.

## Data Availability

The raw data supporting the conclusions of this article belongs to the California Department of Public Health and the participating Tribal Governments and cannot be shared without proper approvals. The readily available public data can be found here: https://library.ucsd.edu/dc/search?f%5Bcollection_sim%5D%5B%5D=California+American+Indian+Tobacco+Initiative+Evaluation+%28CAITIE%29&f%5Bobject_type_sim%5D%5B%5D=data.

## Supplementary material

Supplementary Appendix A Coding List.
