# Supplementary material for: Community dynamics influencing commercial tobacco control policy development on California American Indian lands: a qualitative analysis of baseline CAITIE data
Source: Front Public Health. 2025 Jul 9;13:1464022. doi: 10.3389/fpubh.2025.1464022 (PMC12283582; doi:10.3389/fpubh.2025.1464022)
Supplement: Supplementary Appendix A — Coding List. [file Data_Sheet_1.docx]

**Appendix A: Coding List**

**Code Category 1: Tribal community dynamics favoring new CTC policies**

- Changing social norms

Changing social norms is the code that was used to identify statements of KIs whenever they spoke about the role that CTC policy plays in modifying expectations surrounding commercial tobacco use in public spaces, often slowly over time. Statements that were labeled with this code represented one set of community dynamics favoring new CTC policies. Such statements suggested that policies that change social norms can act as a positive feedback loop and lead to greater acceptance of CTC policies in the future. The theme of changing social norms was expressed by KIs on 21 separate occasions.

- The influence of Tribal leadership

The influence of Tribal leadership is the code that was applied to statements made by KIs that described the unique sway that Tribal leadership over the development of CTC policy. These statements represented the impacts that a given Tribal leader’s own dispositions toward CTC policy can have on new CTC policy development. While these statements were typically made regarding Tribal leadership favoring CTC policy development, Tribal leadership was a recurring theme that occasionally described a community dynamic opposing CTC policy development. The theme of the influence of Tribal leadership was expressed 38 times by KIs.

**Code Category 2: Tribal community dynamics opposing new CTC policies**

- Respect for autonomy

Respect for autonomy is the code that was applied to statements made by KIs that illustrated the value placed on personal freedom. Statements that received this code represented how important the ability to do what one chooses with one’s own body without infringement was to the Tribal community in question. The theme of respect for autonomy was expressed by the KIs 11 times.

- Preference for ‘small-p’ policies

Preference for 'small-p' policies is the code that was applied to statements made by KIs that expressed the ways in which CTC policy was upheld through mutual accountability and social pressure, rather than through punitive measures. Statements that received this code represented how Tribal communities preferred suggestive enforcement of CTC policy, and how that approach to enforcement may still be effective in reducing the public health harms of commercial tobacco use. The theme of preference for ‘small-p’ policies was articulated 27 times by KIs.

- Concern for lost revenue

Concern for lost revenue is the code that was applied to statements made by KIs that represent the worry that CTC will reduce the revenue that a given Tribe may be able to bring in, either through Tribal gaming/ casinos, or through the sales of commercial tobacco products in Tribally owned markets. The theme of concern for lost revenue was expressed 15 times by KIs.
